# Reasons why Mothers Choose Human Milk as Their Method of Infant Nutrition: A Mixed Methods Systematic Review Protocol

**DOI:** 10.1177/18911803261462950

**Published:** 2026-06-19

**Authors:** Niamh Ryan, Patricia Leahy Warren, Siobhain M O’Mahony, Helen Mulcahy, Lloyd Frank Philpott

**Affiliations:** 1 School of Nursing and Midwifery, 8795 University College Cork, Cork, Ireland; 2 Department of Anatomy and Neuroscience, APC Microbiome Ireland, 8795 University College Cork, Cork, Ireland

**Keywords:** human milk, maternal and child health, nutrition, mixed methods systematic review

## Abstract

**Background:**

Human milk is a complex, dynamic, living biological fluid uniquely tailored to meet the nutritional needs of the human species. In addition to this it also has a protective role in health by providing beneficial microbes and prebiotic oligosaccharides that aid in developing the neonatal gut microbiome, and by containing immune molecules that help regulate long-term inflammatory responses. Despite growing evidence of human milk’s composition and benefits, breastfeeding rates remain low in many countries. Some studies suggest that understanding the health benefits and composition of human milk may increase a mother’s motivation to breastfeed or provide human milk. However, first it is necessary to summarize and synthesize the available data on maternal reasons for providing human milk in any form to their infants, to examine the evidence in this area.

**Methods:**

A mixed method systematic review will be conducted including qualitative, quantitative, and primary mixed-methods studies that explore the reasons why mothers choose breastmilk as their method of infant nutrition. The PICo framework will inform the search strategy including five databases CINAHL Complete (EBSCOhost), Medline (PubMed), Web of Science and Scopus (Elsevier) from inception to date of searching. Following screening the quality of the studies will be assessed using the standardized JBI critical appraisal tools, selected based on each study’s methodology. Data extraction will follow the JBI mixed methods data extraction form, and will involve data transformation, synthesis, and integration. This systematic review will adopt a convergent integrated approach in line with JBI guidelines.

**Protocol Registration:**

Registered with Prospero (CRD42024586984).

## Background

Human milk is a complex, dynamic nutritional source recommended exclusively for all infants up to six months of age, and in combination with solid foods until two years or beyond (HSE, 2019; Meek et al., 2022; WHO, 2024b). Breastfeeding provides immediate benefits, including lower risks of infections such as necrotizing enterocolitis, respiratory illnesses, and sudden infant death syndrome, as well as long-term advantages, including improved neurodevelopment and reduced risk of obesity, cardiovascular disease, and diabetes in adulthood (Eidelman et al., 2012; Horta et al., 2023; Horta et al., 2015; Horta & Victoria, 2013). Beyond infant health outcomes, breastfeeding and human milk also confer important benefits for mothers, including reduced risk of breast and ovarian cancer and improved cardiometabolic health, as well as broader societal benefits such as reduced healthcare costs and environmental sustainability (Victora et al., 2016).

The health benefits of human milk derive from the highly diverse composition of this living biological fluid (Ballard & Morrow, 2013). In addition to supplying essential nutrients for infant health, it contains, leukocytes, hormones, microRNA and stem cells along with pioneering microbes and prebiotic oligosaccharides that aid in establishing the neonate’s gut microbiome (Baumgartel et al., 2023; Di Benedetto et al., 2020; Hatmal et al., 2022; Milani et al., 2017). The balance of the human gut microbiome is crucial for overall well-being, with disruptions linked to increased risk to long term health problems such as cardiometabolic diseases, digestive and neurological disorders (Codagnone et al., 2019; Hantsoo & Zemel, 2021; Mafra et al., 2014; Mohammadkhah et al., 2018; Moloney et al., 2014; Robertson et al., 2019; Zafar & Saier, 2021; Zheng et al., 2020). Furthermore, cytokines and immunoglobulins, among other immune molecules in human milk, play key roles in the development of the infant’s immune system and inflammatory responses responsible for long term health (Baumgartel et al., 2023; Lodge & Dharmage, 2016; Royal & Gray, 2020). Therefore, there is robust evidence which indicates that human milk cannot be considered as simple nourishment but the transmission of its biofactors represents a significant predictor for the future health of both a lactating mother and a newborn (Verduci et al., 2021).

Provision of Human milk is vital for achieving Sustainable Development Goal (SDG) target 3.4 of reducing premature mortality from non-communicable diseases by one-third by 2030 and contributing significantly to Goal 3 of ensuring healthy lives and well-being for all (United Nations, 2022). It is also recognised within global public health policy frameworks, including the Sustainable Development Goals and World Health Organization infant feeding targets, which aim to support infant feeding decision-making and improve breastfeeding practices at population level. Human milk feeding is therefore a key component of international targets to increase the rate of exclusive breastfeeding to at least 50% in the first 6 months by 2025, and to 70% by 2030 (WHO, 2024a, 2024b). However, despite the well-documented health benefits for both newborns and mothers, breastfeeding rates remain low in many countries including Ireland which has one of the lowest rates of breastfeeding in the world with 63% of babies receiving human milk at birth and <5% exclusively breastfeeding at 6 months (WBTi, 2023). It is essential to recognize that it may not always be feasible or adequate for every mother to breastfed (Quebu et al., 2023). Exclusive breastfeeding (EBF) faces multiple barriers, including unemployment, caesarean deliveries, lack of EBF awareness, and conflicting family advice (Ayodeji Sokan-Adeaga et al., 2019; Behzadifar et al., 2019; Quebu et al., 2023). Other obstacles reported include the mother’s return to full-time work after maternity leave, as well as social and cultural beliefs and practices (Leahy-Warren et al., 2017; Paul et al., 2024; Philip et al., 2023). Promoting maternal health initiatives within the healthcare delivery agenda is essential for encouraging exclusive breastfeeding (Quebu et al., 2023). To be effective, however, it requires coordinated efforts from the community, government, and institutions to shift the responsibility away from the mother alone.

Increased awareness and knowledge of the benefits of human milk/breastfeeding has been advocated by recent studies in Ireland to improve initiation and duration rates (Philip et al., 2023). Some studies suggest that understanding the health advantages of human milk can increase a mother’s desire to lactate and can prolong the duration of breastfeeding (Adda et al., 2020; Thepha et al., 2018). There is also some literature suggesting a maternal perception that formula is as beneficial as breastfeeding, identifying possible knowledge deficits in the area (Quinn et al., 2023; Radzyminski & Callister, 2016). Initial screening identifies a small number of studies exploring reasons given for breastfeeding, at present there is no existing or on-going mixed methods or individual systematic reviews on the subject. Considering the expanding research in human milk over the last number of years, it is currently unclear whether the correct knowledge of the benefits or awareness of the composition of human milk plays a role in influencing a mother’s reasons to breastfeed or the duration of breastfeeding. Therefore, conducting a systematic review is justified to provide a critically appraised and synthesized answer to this specific review question (Munn et al., 2018). A mixed-methods systematic review (MMSR) is proposed to explore and integrate quantitative, qualitative and primary mixed-methods studies, offering a comprehensive understanding of the literature available including both statistical outcomes and contextual insights (Stern et al., 2021). The aim of this review therefore is to identify and synthesis the primary reasons why mothers choose human milk as their method of infant nutrition.

## Objectives

This mixed method systematic review (MMSR) will be conducted in accordance with the Joanna Briggs Institute (JBI) methodology guidance for conducting Mixed method systematic review (MMSR) (Aromataris & Munn, 2024). The review can be addressed by both qualitative and quantitative research designs therefore a convergent integrated approach will be used (Lizarondo et al., 2022). The review was guided by the PRISMA guidelines and is registered on PROSPERO (CRD42024586984). The aim of this review is to systematically evaluate the evidence on the reasons why mothers choose human milk as a method of infant nutrition, incorporating both qualitatively described and quantitatively reported reasons. The objectives of the review were:

(1) How do mothers describe their reasons for choosing human milk as a method of infant nutrition?
(2) What reasons are reported by mothers in quantitative research tools when choosing human milk as a method of infant nutrition?

## Methods

### Inclusion Criteria

This MMSR protocol follows a PICo framework (Population, Phenomenon of Interest, Context) as recommended by JBI (Stern et al., 2021). The review will be reported in accordance with the Preferred Reporting Items for Systematic Review and Meta-Analysis Protocols (PRISMA-P) statement (Supplemental file 1). The PICo framework is presented in the table below (Table 1), with the inclusion and exclusion criteria linked to each concept.

**Table 1:** PCC Framework

PICo framework	​	Inclusion	Exclusion
Population	Mothers who give their infants human milk.	Mothers who are breastfeeding or who has breastfed. This included any attempt to iniate breastfeeding, or the expression of breastmilk. Mothers who combination feed will be included if there is a discussion regarding reasons for human milk as part of their nutrition. Mothers who give medically directed donor banked milk or non-gestational parents milk if choice for human milk can be extracted	Babies who are formula fed.
Phenomena of interest	Reasons for choosing human milk	Reason around choice for human milk in any form Reasons around choice for breastfeeding methods, if data can be extracted on choice of breastmilk/human milk.	Focus on experiences/attitudes of breastfeeding without exploring reasons. Focus on the reasons for breastfeeding as a method that are related to maternal wellbeing and mother–infant bonding etc, without discussing human milk. Breastfeeding barriers or reasons for cessation to breastfeed.
Context	Global maternal and child health.	Global perspectives across acute and primary care services.	None

### Population

This review will consider studies that include mothers who undertook any duration of breastfeeding or expression of human milk and used it for infant nutrition including medically directed donor milk. It will also include mothers who used expressed breast milk and those who undertook combination feeding if there is a discussion on reasons for intention to use breastmilk in some context. Studies focused on milk bank donation or milk sharing is excluded unless there is a discussion regarding maternal reasons for use of human milk in the data.

### Phenomena of Interest

The main area of interest is the reasons for choice, decision or intention to use breastmilk. Studies exploring the choice for breastfeeding with be included to not miss valuable data. It will not focus on experiences or barriers or reasons for cessation of breastfeeding.

### Context

The context is in the theme of global maternal and child health across any range of acute and primary care settings.

### Types of Studies

This MMSR will include qualitative, quantitative and primary mixed methods study designs for inclusion. Eligible quantitative studies will include those reporting descriptive or inferential data relating to maternal reasons for choosing human milk feeding, including survey responses, frequency of reported motivations, attitudinal measures, and analyses of factors associated with feeding decisions Mixed methods studies are only included if it is possible to clearly extract data from the quantitative or qualitative components as this allows disaggregation to occur (Stern et al., 2021). Previous reviews will be included and interrogated to find the primary studies relating to the inclusion and exclusion criteria.

Table 2 identifies the full eligibility criteria.

**Table 2:** Eligibility Criteria

Included	Excluded
**Primary research:** Experimental and observational studies: • Quasi- experimental • Cohort studies • Case control • Cross sectional • Case series Qualitative studies	Primary mixed methods studies where data is presented as integrated results Secondary analysis Pilot studies
**Secondary research:** Systematic reviews and meta- analysis, meta synthesis	**Secondary research:** Narrative reviews
Years of publication: ALL	NA
​	Grey literature or unpublished literature including case reports, book chapters, guidelines, commentaries, editorials, letters to the editors
English	Non – English

This MMSR will be conducted in accordance with the JBI methodology guidance for conducting MMSRs as outlined in Chapter 8: Mixed methods systematic reviews in the JBI Manual for Evidence synthesis (Aromataris & Munn, 2024). This review question can be addressed by both qualitative and quantitative research designs therefore a convergent integrated approach is advised by JBI guidance (Lizarondo et al., 2022). This process includes data transformation converting quantitative data into textual descriptions and enables reviewers to merge qualitative and quantitative data (Aromataris & Munn, 2024). The search will be restricted to English to avoid interpretation anomalies. Systematic reviews are excluded as primary studies are more favourable to extract the primary data.

### Search Strategy

The review will follow the recommended three-step JBI process along with guidance from Campbells searching guideline. (Aromataris & Munn, 2024; MacDonald et al., 2024) First, initial searches will be carried out in PubMed and CINAHL to identify relevant studies. Following this, text words, keywords, and index terms from the identified articles will be examined to develop a comprehensive search strategy for all databases. (See Supplemental File 2). The keywords and search terms will be peer-reviewed using PRESS guidance with a librarian. All searches will utilize Boolean operators (AND, OR) in title (TI) and abstract (AB) searches, with CINAHL Headings and MeSH headings applied where appropriate. The following databases will be used CINAHL Complete (EBSCOhost), Medline (PubMed), Web of Science and Scopus (Elsevier) from inception to date of searching. There will also be hand searching of specific journals related to this subject such as British journal of midwifery, International Breastfeeding Journal and BMC pregnancy and childbirth. Finally, the reference lists for all studies selected will be reviewed for additional studies which may have not be previously included. The review will restrict analysis to English articles; however initial searches will include all languages to determine the number if any in other languages to avoid bias.

### Study Selection

After completing the search, all selected studies will be exported to Covidence, and duplicates will be removed. The papers are then screened by two reviewers (NR HM LP SOM PLW) in 2 stages: title and abstract screening, and full-text screening. Any exclusion of an article at title-screening stage will require two reviewers. The full text of selected papers will be assessed against the full inclusion criteria, also by two reviewers. The reasons for excluding sources of evidence at the full-text stage that do not meet the inclusion criteria will be documented and reported in the review. Any conflicts during the screening process will be resolved by involving a third reviewer (Aromataris & Munn, 2024). The results of the search will be presented in a Preferred Reporting Items for Systematic Reviews and Meta-Analyses (PRISMA) flow diagram (Page et al., 2021).

### Assessment of Methodological Quality

The remaining studies included will be appraised for methodological quality by two independent reviewers using the standardised critical appraisal instruments from JBI SUMARI, depending on their study design (Aromataris & Munn, 2024). Any disagreements that occur between the reviewers will be resolved through discussion and agreement with a third reviewer. Study authors will be contacted to seek clarification or to request any missing data if required (Aromataris & Munn, 2024). All the studies regardless of their methodological quality will undergo extraction and synthesis however the quality of the study will be considered at the time of data analysis. Following the appraisal, the results will be documented in a narrative descriptive format and visually presented in a table.

### Data Extraction

Two independent reviewers will extract data from all studies included in the review using a standardized JBI data extraction form for MMSR, following a convergent integrated approach (Aromataris & Munn, 2024). This will undergo a pilot test by two independent reviewers on 3 studies (One qualitative, one quantitative and one mixed method if available). The extracted data will include information relating to the PICo i.e. the population divided into breastfeeding mothers, mother who express milk or provide donor milk and mother who combination feed in their choice of use of human milk as the phenomena of interest. The context of the studies if in primary or acute care across the globe will be extracted. Maternal characteristics (socioeconomic status, age, ethnocultural groups, number of children, supports etc.) that are provided will also be extracted along with the study year, design, methods, sampling, inclusion and exclusion criteria, study setting, geographic location and any findings that relate to the review question. The quantitative data will consist of the results from statistical tests conducted, which may include both descriptive and inferential statistics, depending on the nature of the data (Aromataris & Munn, 2024). The credibility of the qualitative data will be assessed based on how well the findings align with the identified themes and/or subthemes (Aromataris & Munn, 2024). JBI guidance outlines three levels of credibility: unequivocal, credible, and not supported (Aromataris & Munn, 2024).

### Data Transformation

In this phase the quantitative data will be translated into “qualitized” data. For example, this may involve narratively phrasing the responses of scales and surveys completed with mothers in the topic areas. The qualified data is then processed in a thematic analysis form which allows the MMSR to examine the degree of concordance between the qualitative and quantitative data (Stern et al., 2021). The Joanna Briggs Institute (JBI) recommends this approach, as it is considered less prone to errors then attributing numerical value to qualitative data (Stern et al., 2021).

### Data Synthesis and Integration

The MMSR convergent integrated methodology from JBI guides the approach to data synthesis and integration. It involves combining the transformed, qualitized data with the qualitative data (Aromataris & Munn, 2024). Thematic synthesis will be completed involving the systematic coding of all the data which will have grouping codes based on similarities to create a summary of findings (Stern et al., 2021).

## Supplemental Material

Supplemental Material - Reasons why Mothers Choose Human Milk as Their Method of Infant Nutrition: A Mixed Methods Systematic Review Protocol Supplemental Material for Reasons why Mothers Choose Human Milk as Their Method of Infant Nutrition: A Mixed Methods Systematic Review Protocol by Niamh Ryan, Patricia Leahy Warren, Siobhain O’Mahony, Helen Mulcahy, Lloyd Philpott in Campbell Systematic Reviews.

Supplemental Material - Reasons why Mothers Choose Human Milk as Their Method of Infant Nutrition: A Mixed Methods Systematic Review Protocol Supplemental Material for Reasons why Mothers Choose Human Milk as Their Method of Infant Nutrition: A Mixed Methods Systematic Review Protocol by Niamh Ryan, Patricia Leahy Warren, Siobhain O’Mahony, Helen Mulcahy, Lloyd Philpott in Campbell Systematic Reviews.
