# Supplementary material for: Reasons why Mothers Choose Human Milk as Their Method of Infant Nutrition: A Mixed Methods Systematic Review Protocol
Source: Campbell Syst Rev. 2026 Jun 19;22(2):18911803261462950. doi: 10.1177/18911803261462950 (PMC13294536; doi:10.1177/18911803261462950)
Supplement: Supplemental Material - Reasons why Mothers Choose Human Milk as Their Method of Infant Nutrition: A Mixed Methods Systematic Review Protocol [file sj-pdf-2-cam-10.1177_18911803261462950.pdf]

**Date**	13.08.24 – CINAHL SEARCH 1	
**Research Topic**	**Reasons why mothers choose human milk as their method of infant nutrition: A systematic review.**	
**Search Strategy**	**Key concepts**	**Synonyms/alternative terminology (consider regional variations here also) – combine using OR**
	Reason*OR Cause* OR driver* OR motive* OR motivations OR determinant* OR justification OR rationale	
AND		
	Choice OR Choose OR Decision* OR Decide OR option* OR decision-making OR “decision making” OR preference* OR [illegible]ntention*	
AND		
	Mother* OR maternal Or woman OR women Or female OR MH “Mothers”	
AND		
	Breastmilk OR BM OR OR “Human Milk” OR Breastfed Or colostrum OR Breastfeeding OR breast-feeding OR lactating Or Lactation OR Milk OR MH “Milk,Human +” OR “MH “Breast feeding+”	
AND		

S1	TI (Reason*OR Cause* driver* OR motive* OR motivation* OR determinant* Or rationale OR justification) OR AB (Reason*OR Cause* driver* OR motive* OR motivation* OR determinant* OR rationale OR justification)	112599	
S2	TI (Choice OR Choose OR Decision* OR Decide OR option* OR decision-making OR “decision making” OR preference* OR Intention*) OR AB (Choice OR Choose OR Decision* OR Decide OR option* OR decision-making OR “decision making” OR preference* OR Intention*)	480225	
S3	TI (Mother* OR maternal OR women OR woman OR female) OR AB (Mother* OR maternal OR women OR woman OR female)	606,836	
S4	(MH "Mothers+")	58,799	
S5	TI (breastmilk OR human milk OR BM OR Breastfed OR colostrum OR Breastfeeding OR breast-feeding OR lactating or Lactation) OR AB (breastmilk OR human milk OR BM	38,188	

	OR Breastfed OR colostrum OR Breastfeeding OR breast-feeding OR lactating or Lactation)		
S6	(MH "Milk, Human+")	8574	
S7	(MH "Breast Feeding+")	28,149	
S8	TI (infant nutrition* OR infant feed* OR infant food* or infant diet* OR diet OR nutrition) OR AB (infant nutrition* OR infant feed* OR infant food* or infant diet* Or diet OR nutrition)	12,736	
S8	S3 & S4	618803	
S9	S6 OR S7	48,515	
S10	S1 AND S2 AND S8 AND S9	147	

See updated search
